# High-Precision Lens-Less Flow Cytometer on a Chip

**DOI:** 10.3390/mi9050227

**Published:** 2018-05-10

**Authors:** Yuan Fang, Ningmei Yu, Yuquan Jiang, Chaoliang Dang

**Affiliations:** 1 School of Automation and Information Engineering, Xi’an University of Technology, Xi’an 710048, China; fangyuanmy@163.com (Y.F.); yqjiang@xaut.edu.cn (Y.J.); dangclkk@163.com (C.D.); 2 School of Electrical and Electronic Engineering, Baoji University of Arts and Sciences, Baoji 721016, China

**Keywords:** cell analysis, lens-less, microfluidic chip, twin-image removal, POCT

## Abstract

We present a flow cytometer on a microfluidic chip that integrates an inline lens-free holographic microscope. High-speed cell analysis necessitates that cells flow through the microfluidic channel at a high velocity, but the image sensor of the in-line holographic microscope needs a long exposure time. Therefore, to solve this problem, this paper proposes an S-type micro-channel and a pulse injection method. To increase the speed and accuracy of the hologram reconstruction, we improve the iterative initial constraint method and propose a background removal method. The focus images and cell concentrations can be accurately calculated by the developed method. Using whole blood cells to test the cell counting precision, we find that the cell counting error of the proposed method is less than 2%. This result shows that the on-chip flow cytometer has high precision. Due to its low price and small size, this flow cytometer is suitable for environments far away from laboratories, such as underdeveloped areas and outdoors, and it is especially suitable for point-of-care testing (POCT).

## 1. Introduction

Cell analysis using an optical microscope or a flow cytometer is an important technique in biology and medicine [1]. Optical microscopes can obtain focus images of cells for biomedical applications, and flow cytometers can collect the signature of a large number of cells in liquid specimens with high analysis speed. However, these instruments are unsuitable for outdoor and undeveloped areas because of their high price and large size. Currently, there is a need for a small and inexpensive cell analysis device that combines the properties of the above two devices.

Over the past decade, lens-less imaging has been considered a good way to reduce the volume and cost of cell analysis tools. Seung Ah Lee and Guoan Zheng designed opto-fluidic microscopes using a complementary metal oxide semiconductor (CMOS) image sensor (CIS) and a microfluidic channel [2,3,4,5,6,7]. To weaken the shadow-imaging diffraction, the distance between the cells and the surface of the image sensor must be shorter than 2 μm. These researchers mounted a micro-channel on a CIS by removing the protective glass and Bayer filter. To improve the spatial resolution of cell images obtained with a 4× object lens, they used a multi-frame, super-resolution algorithm based the sub-pixel movement of cells flowing through the micro-channel. At the same time, Aydogan Ozcan and Serhan O. Isikman designed numerous lens-free on-chip microscopes based on incoherent digital holography [8,9,10,11,12,13,14,15,16,17,18,19,20,21,22,23,24,25]. The lens-free on-chip microscopes capture digital diffractive images of cells by using an in-line holographic structure. The diffractive images were used to reconstruct clear images of the cells using angular spectrum theory [26], and the resultant clear cell images are comparable to those obtained by a 10× object lens with a numerical aperture of ~0.1–0.2. Later, Se-Hwan Paek and Sungkyu Seo proposed a new method to classify different types of cells using digital diffractive images [27,28,29,30]. Mei Yan and Hao Yu conducted a blood cell analysis with a single-frame super resolution [31]. The concept of a lens-less microscopy technique is a novel idea for the miniaturization of flow cytometry, but the accuracy and speed of cell counting in such a method are challenges. At present, most devices based on a lens-less platform use only one frame to count cells, and this leads to inaccurate cell counting [27]. It is not easy to distinguish between cells and dust using a static image, which has a great influence on the ability to count with high precision.

In this manuscript, we propose an on-chip flow cytometer system based on lens-less imaging and a microfluidic control technique to improve the speed of cell analysis. The system causes cells to flow through a micro-channel in a polydimethylsiloxane (PDMS) microfluidic chip above a CIS. A near-coherent light source is mounted above the microfluidic chip (~5 cm), and diffraction shadow images of cells generated by the near-coherent light source are then captured by the CIS. To obtain clear images of cells, a phase iterative reconstruction algorithm is used for image diffraction [32]. In addition, the system can obtain a very accurate image without cells absented for background removal. After the background is removed, images of each segmented cell can be acquired from the whole image more precisely. Therefore, we can more accurately extract features from each cell image and quickly classify and count cells.

Because of the low intensity of near-coherent light caused by a pinhole, the exposure time of the image sensor in the system is longer than 400 ms. Therefore, there is stronger motion blur while the cells are quickly flowing in the micro-channel. To solve this problem, this manuscript proposes a method in which the cells in the micro-channel are imaged simultaneously in a large field of view (FOV) instead of with a flow cytometer method in which the cells pass through the testing area at high speed. In other words, the method takes advantage of the larger FOV of the CIS to reduce the cell flow velocity. To utilize the large FOV of the CIS, we design an “S” channel shape. As a result, we can ensure that the CIS captures the maximum possible number of cells in a frame. In addition, the cells in current frame flow out of the micro-channel completely before the next exposure of the CIS. Thus, all the cells in each frame are new cells, and the cells in each individual frame can be evaluated to increase the number of tested cells. Regarding cost, the CIS is commonly used in industrial cameras and mobile phones, so the price is very low (below $10). The microfluidic chip comprises a PDMS channel and a piece of thin glass (0.18 mm), making it very cheap and easy to replace. Overall, this manuscript proposes an on-chip cytometer that can test blood cells, bacteria, and other micro-particles in liquids. Because of the low price and small volume, the system is especially suitable for places far away from the laboratory and undeveloped areas and for family health tests.

## 2. Materials and Methods

### 2.1. System Setup

**Figure 1:**
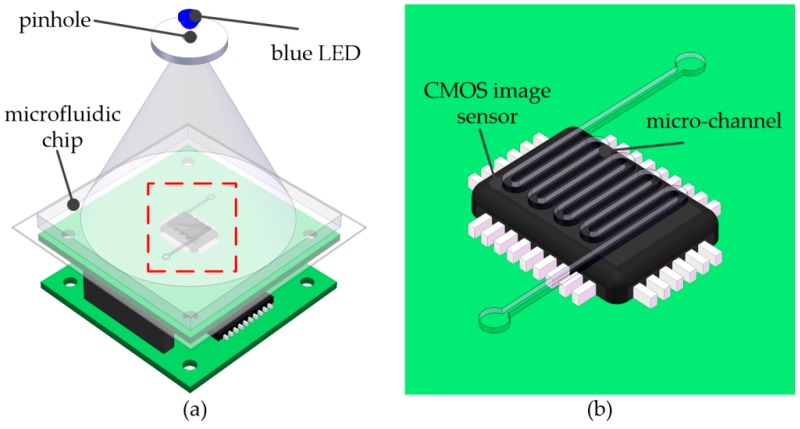
The structure of an on-chip flow cell counting system: (**a**) The general structure of the system; (**b**) the micro-channel on the image sensor (CIS) surface in the red box in (**a**).

The flow cytometer utilized a lens-less imaging technique based on an in-line holography structure, and the overall structure is shown in Figure 1.

As shown in Figure 1, the flow cytometer comprised a greyscale CIS (Aptina MT9P031, Micron Technology, Pennsylvania, ID, USA), a PDMS microfluidic chip and a blue light-emitting diode (LED) light source (central wavelength of ~465 nm). The pixel size of the CIS was 2.2 μm, the effective pixel size was 2592 H × 1944 V (5.7 mm × 4.2 mm), and the imaging area reached ~24.4 mm^2^. To obtain holographic diffraction patterns on the surface of the CIS, the blue light LED was located 5 cm above the surface of the image sensor. In addition, there was a plate with a pinhole (diameter of 0.1 mm) at the front of the LED to obtain a coherent light source. To utilize the large FOV of the CIS, an S-type micro-channel was designed that could easily determine the volume of liquid samples and count the maximum possible number of cells in a frame. Moreover, the concentration of cells in a specimen could be calculated accurately, similar to a classic cell counting chamber. We used a PDMS channel and a piece of thin glass bonded together to obtain a microfluidic chip to capture the holograms of cells (the diffractive shadow images of cell) and fix the microfluidic chip on the surface of the CIS. We briefly introduce the fabricated process of the microfluidic below.

The photoresist (SU-8 2015, Microchem, Westborough, MA, USA) and a silicon wafer (4 inches in diameter) were used to fabricate positive model. The 3 mL of photoresist was dropped in the centrality of a wafer, and the photoresist film was 30 μm in thickness after using the spin coater at 1500 r/min for 15 s. Then, the silicon wafer was pre-baked for 15 min at 95 °C. The pre-designed channel photolithography plate was used for exposure on the lithography machine for 125 s. Next, the exposed wafer was after-baked for 3 min at 95 °C, and developed for 3 min. Then, we poured 30 g of liquid PDMS on the positive film, and put it in baking box for 40 min at 95 °C to solidify. The solidified PDMS layer and a piece of thin glass were bonded by vacuum plasma technique. Finally, the PDMS layer was drilled the holes of the inlet and outlet to finish the microfluidic chip.

**Figure 2:**
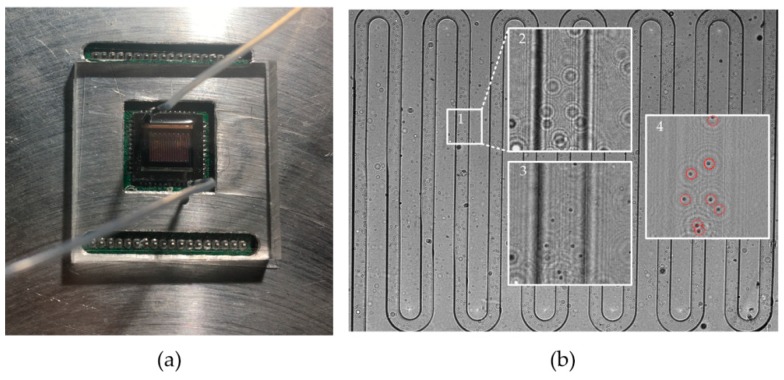
The proposed flow cytometer: (**a**) The flow cytometer system; (**b**) the holograms of the microfluidic chip captured by the system. Box 2 is an amplificatory image of box 1, and box 3 is a diffractive reconstruction image of box 2. Box 4 is the image of box 3 with the background removed, where the red circles mark the cells.

However, since a microfluidic chip was used, a cell sample could be continuously detected, similar to a flow cytometer, as shown in Figure 2.

Next, we prepared an experimental platform to obtain the features and parameters of the proposed system. In addition, we found that the exposure time of the image sensor in this system was greater than 400 ms. Unfortunately, motion blur is caused by the movement of cells in the sample when the image sensor is operating during the exposure time. Therefore, we considered that instead of the cells flowing through the detection area at high speed, a large number of cells passed through the exposure region at one time. In other words, the system utilized the large FOV of the CIS to obtain a large number of images of cells from each frame. To avoid the motion blur caused by cell flowing, we used a method of periodically controlling the flow velocity of the specimen. There was only one inlet and one outlet in the micro-channel, ensuring that the flow of all the tested cells out of the micro-channel and that of the new cells flow into the micro-channel took a short time. To obtain a sufficient processing time for the image processing algorithm, the new cells were injected into the micro-channel during the image processing period. Subsequently, all the tested cells flowed out the micro-channel, and then the flow of the cells stopped and the cell images were captured by the image sensor. With several repetitions, the device was able to collect the maximum possible number of cell signatures to improve the accuracy of the analysis.

### 2.2. Sample Preparation

The flow cytometer is suitable for samples with a large number of cells, such as blood. Therefore, we performed an experiment with whole blood. The concentration range of red blood cells (RBCs) in whole blood is from ~4 × 10^12^/L to 5.5 × 10^12^/L. To ensure the reconstruction of the wavefront in the in-line holography system, we had to reduce the concentration of cells in the whole blood. According to the experiments, we found that 1:400 was a suitable volume dilution to count blood cells. When RBCs were tested, the dilution ratio was 1:400, corresponding to 10 μL of whole blood diluted with 4 mL of phosphate buffer saline (PBS, 0.0067 M PO_4_), and the resulting solution was pumped into the microfluidic chip for testing. 

The concentration of white blood cells (WBCs) in whole blood is 4 × 10^9^/L–10 × 10^9^/L, and the ratio of WBCs to RBCs is close to 1:1000. When WBCs were tested, 200 μL of a whole blood sample was diluted with 400 μL of RBC lysis buffer and this was then injected into the micro-channel after one minute of delay. The study was approved by the School of Automation and Information Ethics Committee, Xi’an University of Technology.

### 2.3. Reconstruction of Lens-Less Holographic Images

**Figure 3:**
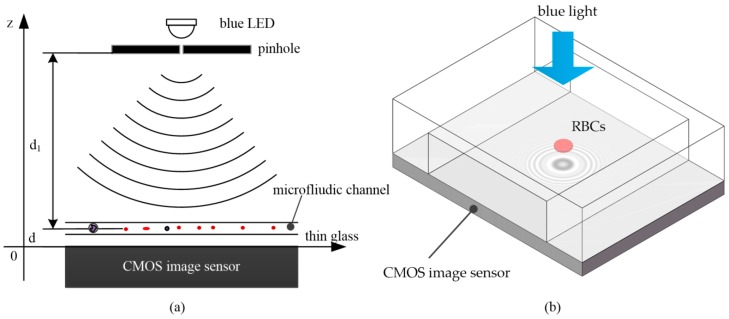
Graphic sketch of an in-line holographic microscope: (**a**) the structure of the lens-less holographic imaging system; (**b**) the procedure for capturing the lens-less hologram by the CIS.

The lens-less imaging technique utilizes an in-line holographic structure proposed by Gabor [33] to reconstruct the image of the cell plane. The lens-less holographic imaging system is mainly composed of a blue LED light source, a pinhole plate, a microfluidic chip and a CIS, as shown in Figure 3.

Because of the infinitesimal size of blood cells (~2–15 μm), the shadows of the blood cells on the surface of the CIS are diffraction images. Due to the influence of diffraction phenomenon, the shorter the wavelength of light source is, the higher the spatial resolution of the microscopic image becomes. In the most commonly used LED of single frequency light sources, a blue light source has the shortest wavelength. So we chose a blue LED as the light source. For convenience, we assumed that the cell plane was the object plane and that the surface of CIS was the image plane. The distance from the pinhole to the object plane was *d*_1_, and the distance from the object plane to the image plane was *d* (*d*_1_ >> *d*). According to the angular spectrum theory of diffraction, we can reconstruct an image of the object plane by recording the image plane. We assumed that the transmittance of the sample was *O*(*x*, *y*) and that the complex amplitude of the wavefront through the object plane was as below:

(1) $$U_{d}\left(x,y\right)=1-O(x,y)$$

Here, the image plane is assumed as the plane of *z* = 0, and the object plane is assumed as the plane of *z* = *d*. According to the Rayleigh–Sommerfeld diffraction theory, the transfer function of light waves in two planes separated by a distance *d* is defined as:

(2) $$H_{d}\left(\varepsilon ,\eta \right)=\{\begin{array}{c} \exp\left[jd\frac{2\pi }{\lambda }\sqrt{1-{\left(\lambda \varepsilon \right)}^{2}-{\left(\lambda \eta \right)}^{2}}\right]\\ 0,\;otherwise \end{array},\;\varepsilon^{2}+\eta^{2}<\frac{1}{\lambda^{2}}$$

Here, *ε* and *η* denote the coordinates of a frequency domain and have been transformed by *x* and *y* into a spatial domain. *λ* is the wavelength of the light source. According to the transfer function, we can obtain the complex amplitude of the image plane:

(3) $$U_{0}\left(x,y\right)=H_{d}^{+}\left[1-O(x,y)\right]$$

where $H_{d}^{+}$ and $H_{d}^{-}$ represent the optical forward and backward propagation operators, which carry out a fast Fourier transform and an inverse fast Fourier transform, respectively, belonging to a convolution operator. *d* denotes the distance of the light propagation; in other words, it is the distance between the object plane and the image plane. + and − denote forward propagation and backward propagation along the *z*-axis, respectively. The light intensity in the holographic plane recorded by the image sensor is the square of the amplitude of the light wave, and the light intensity is as below:

(4) $$I_{0}\left(x,y\right)={\left|U_{{}_{0}}\left(x,y\right)\right|}^{2}$$

In Equation (4), *U*_0_(*x*,*y*) is the complex amplitude of the actual light wave in the image plane, but the image sensor can receive only the light intensity, *I*_0_, and the phase is discarded. Normally, image sensor acquisition of the holographic plane light intensity is a linear process, so the light intensity information collected by image sensor can be expressed as:

(5) $$I_{s}\left(x,y\right)=\alpha +\beta I_{0}\left(x,y\right)$$

The amplitude of the light wave in the object plane can be obtained by the reconstruction of the distance image at the back of the image plane:

(6) $$U_{r}\left(x,y\right)=H_{d}^{+}\left[I_{s}\left(x,y\right)\right]$$

With Equations (1)–(6), we obtain Equation (7):

(7) $$U_{r}\left(x,y\right)=D-O^{*}(x,y)-H_{2d}^{+}\left[O(x,y)\right]+H_{d}^{+}\left\{{\left|H_{d}^{+}\left[O(x,y)\right]\right|}^{2}\right\}$$

**Figure 4:**
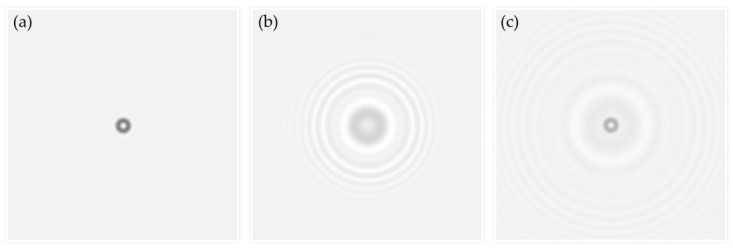
The twin-image problem: (**a**) a 7 μm micro-bead image was used to simulate the reconstruction of lens-less holographic images. (**b**) By simulating the holographic imaging with Equations (1)–(4), we retained the amplitude of the complex number and discarded the phase to simulate the recording procedure of a CIS. (**c**) We used Equation (6) to reconstruct the focus image of object plane, but it was polluted by the twin-image phenomenon.

In Equation (7), the first term is the direct current (DC) component; the second term is the focus image; the third term is the holographic image, which is the focus image backward propagated a distance of 2*d*; and the fourth term is the intermodulation. The second and third terms constitute the twin image, which still appears after the forward transfer reconstruction of the diffraction plane and is difficult to separate. In fact, the twin-image phenomenon, which is caused by the absence of a light phase, is a major problem in the in-line holographic system. In addition, we used micro-bead images obtained with a 10× objective lens to simulate the twin-image problem (Figure 4).

According to Gabriel Koren’s research [32], we can use only one diffractive to reconstruct a focus image of the object plane and suppress the twin-image phenomenon. In our proposed algorithm, only a holographic diffraction image and a cell-absent background image were needed to reconstruct the phase and obtain a focus image of the object plane. The general steps were as follows:

Step 1: Using the square root of the light intensity and the initial value of the phase (generally 0), reversely transfer the diffractive pattern of the image plane back to the object plane by the transfer function to obtain the focus image. However, the initial estimation of the object plane seriously suffers from the twin-image phenomenon. Thus, it is necessary to use the following steps to suppress the twin images. The main operation of the reconstruction process is similar to the frequency domain filter in digital image processing. The transfer function of the filter is shown by Equation (2), and the reconstruction algorithm of the object plane is shown below:

(8) $$U_{r}^{1}\left(x,y\right)=H_{d}^{-}\left[\sqrt{I_{s}\left(x,y\right)}\right]$$

Step 2: The region information of the object is extracted from the preliminary estimated object image, which is used for the object plane constraint. Classic image segmentation algorithms, such as the gradient boundary extraction algorithm and the threshold segmentation algorithm can be used to find the object plane constraint. Because of the low signal-to-noise ratio (SNR) of the image extracted by the CIS, the threshold segmentation algorithm is more reliable. The threshold is 0.34 in this manuscript; in other words, the grey value of cell regions on the object plane is usually less than 0.34.

Step 3: The cell region is the *C* region, and the background is the non-*C* region. Through an iterative algorithm, the cell regions are close to the real image, and the twin-image phenomenon will be weakened on the object plane. The algorithm is

(9) $$U_{r}^{\left(i+1\right)}\left(x,y\right)=\{\begin{array}{c} m\times D\left(x,y\right),x,y\notin C\\ U_{r}^{i}\left(x,y\right),x,y\in C \end{array}$$

where *D*(*x*, *y*) is the background image, which is obtained by the image sensor without cells, and *m* is shown with

(10) $$m=mean\left(U_{r}^{i}\left(x,y\right)\right)/mean\left(D\left(x,y\right)\right)$$

Step 4: The new complex amplitude of the image is obtained by the forward transfer operation. The phase of the newly calculated complex amplitude is retained, and the amplitude is replaced by the original known image plane amplitude. This process is called the image plane constraint:

(11) $$U_{0}^{i}\left(x,y\right)=\left|U_{0}^{1}(x,y)\right|\times \exp\left(j\times \varphi_{0}^{i}\left(x,y\right)\right)$$

The iteration can be completed by repeating the third and fourth steps and can converge after 5–6 iterations. To obtain the missing phase, the algorithm iterates between two planes (object plane and image plane) through the amplitude and makes the iteration convergent using the object plane constraint (Equation (9)) and the image plane constraint (Equation (11)). However, the algorithm converges rapidly in the initial several iterations, and then the convergence is almost stagnant. Furthermore, there is a large error in the estimation of the initial phase when the distance between the object plane and the image plane has a deviation in an actual system. Therefore, the classic phase recovery algorithm is necessary to improve an actual system. The manuscript proposes an initial phase constraint algorithm based on the classic algorithm, in which Equation (8) is replaced by Equation (12):

(12) $$U_{r}^{1}=H_{d}^{-}\left[\sqrt{I_{s}\left(x,y\right)}\times \exp\left(j\times \left(1-\sqrt{I_{s}\left(x,y\right)}\right)\right)\right].$$

In general, there is no linear relationship between the amplitude and phase in a complex number. However, the phase changes of near-coherent light passing through a cell are related to the cell transmittance, and the cell transmittance is also expressed in amplitude. Therefore, there is a weak correlation between amplitude and phase. Using this property, we can estimate the initial phase of the iteration by transmittance. Through the initial phase constraint, the iterative convergent speed is faster, the reconstruction precision is higher, and the anti-jamming ability is stronger.

**Figure 5:**
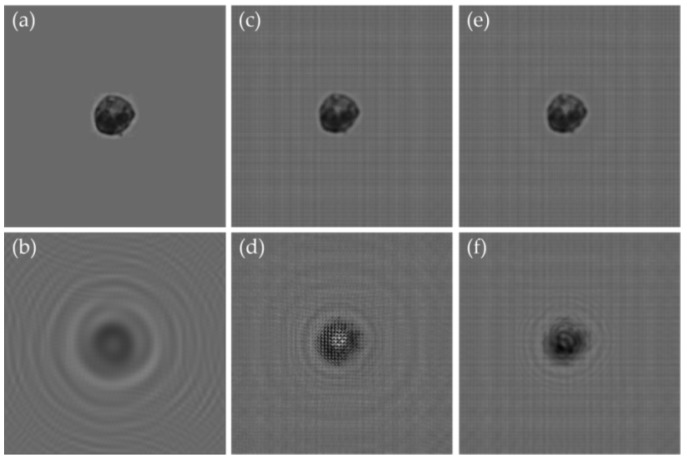
The result of our proposed method and classic method without a phase constraint. (**a**) The image captured by a 20× object lens microscope; (**b**) the diffractive image on the image plane; (**c**) the image reconstructed by Gabriel Koren’s method with 0% deviation in distance; (**d**) the image reconstructed by Gabriel Koren’s method with 20% deviation in distance; (**e**) the image reconstructed by our modified method with 0% deviation in distance; (**f**) the image reconstructed by our modified method with 20% deviation in distance.

To test the performance of the algorithm, we used a dyed leucocyte captured by a 20× object lens microscope to perform a simulation. Using Equations (1)–(5) to establish a diffractive degradation model, we obtained the diffractive pattern of the leucocytes. To replicate our flow cytometer, we chose the same parameters as the actual system for simulation. The central wavelength of the light source was 465 nm, the distance between the object plane and the image plane was 0.875 mm, and the pixel size was 2.2 μm. The iterative algorithm without the initial phase constraint was compared to the iterative algorithm with the initial phase constraint, and the result is shown in Figure 5. 

To test the performance of the two methods, we calculated the root-mean-square error (RMSE) for the reconstructed image of the object plane and original image. Finally, the proposed algorithm was used to reconstruct the cell image on the object plane and compared with the original image to calculate the RMSE:

(13) $$RMSE=\sqrt{\frac{1}{MN}\sum_{m=1}^{M}\sum_{n=1}^{N}{\left(\left|U_{r}^{i}\left(x,y\right)\right|-\left|U_{d}\left(x,y\right)\right|\right)}^{2}}$$

**Figure 6:**
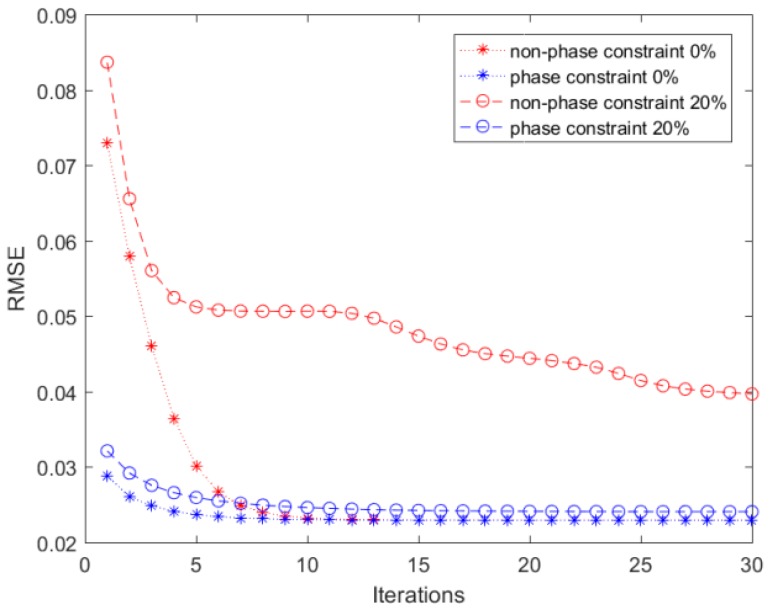
The root-mean-square error (RMSE) of our proposed method and the classic method without a phase constraint.

According to the distance between the object plane and image plane, we conducted two groups of comparative experiments. The first was without deviation, and the second was with 20% deviation. The RMSEs of the two method were calculated by Matlab (Version: 2016b, MathWorks, Endogenous, MA, USA) and are shown in Figure 6.

In Figure 6, the ‘phase constraint’ is our proposed method, and the ‘non-phase constraint’ is the classic method. The proposed method has a faster convergence rate and a lower error rate, making it more conducive to counting and analyzing cells. As shown in Figure 5 and Figure 6, by comparing the two groups with the two methods, we found that the iteration method with initial phase constraints had a faster iteration speed. In the case of a 20% distance deviation, the proposed method was able to restore the cell image, whereas the original method could not restore the image effectively, which has a great influence on the actual system. Moreover, when all the parameters were accurate, the proposed method converged faster, and the RMSE of image reconstruction was smaller. The results in Figure 6 show that our proposed method can greatly reduce the time consumption of the image processing algorithm and provide a guarantee for the real-time implementation of the system.

Finally, we used a frame image of whole blood cells captured by the lens-less flow cytometer to test the computation time. We used Matlab to reconstruct the holographic image, and the hardware was graphics workstation (Xeon E5-2600, 16 GB DIMM DDR4, Intel, Santa Clara, CA, USA). The time consumed for one iteration was about 2 s with the classic method of phase iterative reconstruction, and the proposed method took ~0.1 s longer than the classic method. However, the method we proposed only needed 5 iterations, and the classic method needed 10 times to achieve the same reconstruction effect. Therefore, the time consumed by the propose method was ~12.82 s, and that of the classic one was ~24.42 s. In other words, it means that the proposed method reduced the computational time by ~48%. In general, the two algorithms have almost the same computational complexity. Our algorithm only adds one phase constraint to the first image reconstruction, but its time computation is ~0.19 s.

### 2.4. Blood Cell Analysis Method

**Figure 7:**
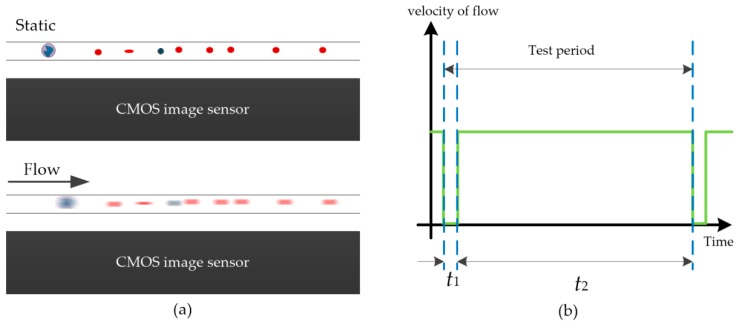
The pulse flow control mode: (**a**) The flow of cells stops in the micro-channel during the CIS exposure time to capture a holographic image of the cells. (**b**) When the system is processing images of cells captured in the last exposure, the tested cells flow out of the micro-channel, and the new cells flow into the micro-channel.

In the on-chip flow cytometer, the blood cells flow in the micro-channel above the image sensor, and their holographic diffraction image is transmitted onto the sensor surface by the near-coherent light source. To reduce the cost and volume of the device, an ordinary blue LED with a limited light intensity was used. The light on the plane of the cells is further weakened because the light is illuminated through a pinhole. Therefore, the exposure time of the image sensor needs to be longer than 400 ms to capture a bright enough hologram. If the blood cells move during the exposure time of the CIS, there is a motion blur, as shown in Figure 7a. To solve this problem, we used a pulse injection method, which is shown in Figure 7b.

In Figure 7, *t*_1_ is the exposure time, and *t*_2_ is the injection time. This process can be controlled by a micro-pump. Due to the high precision of micro-pump control, the injection time and stationary time of the blood cells can be fixed. Therefore, the algorithm can be processed according to fixed parameters. After an experiment, accurate cell image collection and injection of new samples in micro-pump mode can be ensured.

In addition, instead of the micro-pump method, a hand-push model can be used to reduce the cost and volume of the device. In the hand-push model, the motion state of the cells in the microfluidic chip can be detected by the image processing algorithm. The system acquisition accuracy can generally be guaranteed with *t*_1_ > 5 s and *t*_2_ > 20 s. The state of cell motion in the microfluidic chip is detected by the RMSE between two frames.

In addition, there are two important problems, which relate to cell overlap. The first is the cell overlap in the holographic image. As mentioned earlier, the raw image captured by the lens-less platform is a holographic image, so the size of a diffractive image of the cell is ~4 times bigger than that of a focus image. The inevitable cell image overlap was solved by the phase iterative reconstruction algorithm. The other problem relates to the position of cell overlap and the 3D structure of the micro-channel leads to the shadow image overlap. The problem is difficult to solve by digital image processing algorithms. Therefore, we used a diluted cell sample to solve the problem. According to the experiment, the 1:400 dilution ratio is an acceptable ratio for the blood cells, and cell overlap is almost impossible at 1:1000 dilution. Considering the speed of counting, we chose a 1:400 dilution.

The microfluidic chip was mounted above the CIS so that we could easily obtain the background image without cells. In addition, we then injected a fluid sample of cells into the micro-channel to record the holograms and reconstruct the focus images of the cells. The location and size of the cells were determined by threshold segmentation with images with background interference removed. According to our experiment, the flow velocity was 100 μL/min. Because of the infinitesimal volume of the micro-channel (0.246 μL), the digital injection pump was able to replace all cells in the channel less than 1s. However, it took ~15 s to 20 s for the cells in the fluid to become static. Fortunately, were able to use this time to process the cell image. Since the pixel number of the CIS was about 5.04 million, the computer took ~16 s to process the full resolution image.

The on-chip flow cytometry capability of this method, together with its ease of use, may offer a highly precise and lower cost alternative to existing whole blood analysis tools, urine analysis tools and plankton analysis tools, especially for point-of-care biological and medical tests.

## 3. Results and Discussion

To test the performance of our proposed system, we performed an experiment with whole blood cells. In the results section, we show the results of focus image reconstruction for cell counting in our proposed system.

**Figure 8:**
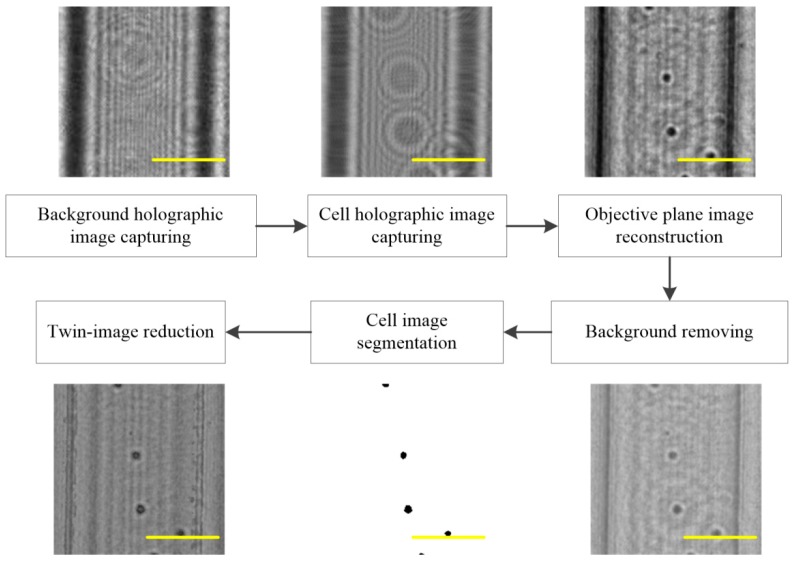
The reconstruction of the focus cell images flow path. To better observe the effect of holographic image reconstruction, which is a small segment of the whole image, all scale bars indicate 100 μm.

Figure 8 shows that the images of the cells were captured by removing the background in the reconstructed image and that the quantity and size of the sample were determined by the image threshold segmentation algorithm. The system obtained an accurate background image, effectively removed the background effect, accurately acquired the location of the sample, and greatly improved the counting accuracy. Finally, the cell concentration was calculated based on the number of cells and the volume of the micro-channel. The micro-channel was 30 μm in height, 150 μm in width, and ~54.6 mm in length; thus, it was easy to calculate its volume as ~0.246 μL and projective area as 8.19 mm^2^.

**Figure 9:**
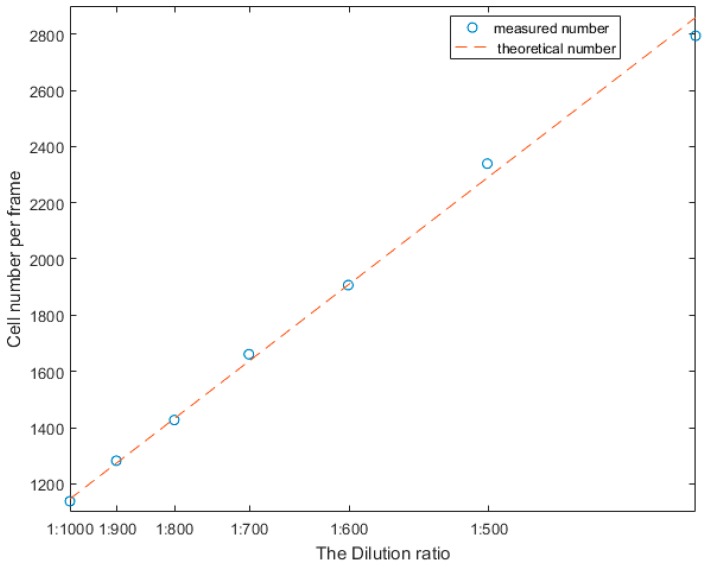
The result of linearity of different concentrations.

Then, we used different concentrations of the whole blood samples to test the linearity and the accuracy of the proposed method. We diluted seven groups of different concentrations of blood cells with a dilution ratio to perform an experiment. The results of this experiment are indicated in Figure 9.

Ultimately, we used whole blood cells to count RBCs and WBCs in order to verify the effectiveness of the cell counting procedure. According to the above preparation method, diluted whole blood cells and lysed whole blood cells were divided twice. In addition, we needed to substract the concentration of WBCs from the concentration of whole blood cells to get the concentration of RBCs. Then, the test results of the proposed system were compared with those of an automatic blood cell analyser (BC-5180, Mindray, Shenzhen, China), and the results are shown in Table 1.

Table 1 shows that the average fractional errors of RBC and WBC counting were 2% and 1.6%, respectively. In other words, the relative error between the proposed system and the whole blood cell counter was less than 2%. This accuracy indicates the potential for applying the proposed system to the early detection of some liquid samples, such as blood tests, urine tests, semen tests, and microbiological tests. In areas where the use of large-scale, high-precision instruments is inconvenient, such as the outdoors, the battlefield, and underdeveloped areas, the tool proposed here can enable early and real-time detection.

## 4. Conclusions

The current paper improved the twin-image recovery algorithm in a coaxial holography system and increased the convergence speed of the iterative algorithm. In addition, this paper proposed a flow cytometer based on lens-less holographic microscopy that improved the counting accuracy to 2.3%. The on-chip flow cytometer based on a pulse injection method can continuously count cells and continuously collect a large number of cell images for subsequent cell analysis. Ultimately, this on-chip flow cytometer is very suitable for use in underdeveloped areas and areas far away from the laboratory because of its low price and tiny size and is in full compliance with the current development trend of point-of-care testing (POCT).

## Figures and Tables

**Table 1:** The result of different sample concentrations.

Sample No.	Tools	White Blood Cells (WBCs) Concentration	Error	Red Blood Cells (RBCs) Concentration	Error
1	Our system	6.83 × 10^9^/L	1.4%	5.22 × 10^12^/L	2.4%
	BC-5180	6.93 × 10^9^/L		5.35 × 10^12^/L	
2	Our system	4.80 × 10^9^/L	1.2%	5.28 × 10^12^/L	1.5%
	BC-5180	4.86 × 10^9^/L		5.36 × 10^12^/L	
3	Our system	6.33 × 10^9^/L	0.6%	4.64 × 10^12^/L	1.9%
	BC-5180	6.29 × 10^9^/L		4.73 × 10^12^/L	
4	Our system	5.10 × 10^9^/L	2.3%	4.61 × 10^12^/L	1.5%
	BC-5180	5.22 × 10^9^/L		4.68 × 10^12^/L	
5	Our system	6.78 × 10^9^/L	2.6%	4.87 × 10^12^/L	2.7%
	BC-5180	6.96 × 10^9^/L		4.74 × 10^12^/L	
